# Cross-Layer Cluster-Based Energy-Efficient Protocol for Wireless Sensor Networks

**DOI:** 10.3390/s150408314

**Published:** 2015-04-09

**Authors:** Aboobeker Sidhik Koyamparambil Mammu, Unai Hernandez-Jayo, Nekane Sainz, Idoia de la Iglesia

**Affiliations:** DeustoTech, Department of Engineering, University of Deusto, Bilbao 48007, Spain;

**Keywords:** MAC, cluster, WSN, clustering, sensor, Aggregation, routing, energy

## Abstract

Recent developments in electronics and wireless communications have enabled the improvement of low-power and low-cost wireless sensors networks (WSNs). One of the most important challenges in WSNs is to increase the network lifetime due to the limited energy capacity of the network nodes. Another major challenge in WSNs is the hot spots that emerge as locations under heavy traffic load. Nodes in such areas quickly drain energy resources, leading to disconnection in network services. In such an environment, cross-layer cluster-based energy-efficient algorithms (CCBE) can prolong the network lifetime and energy efficiency. CCBE is based on clustering the nodes to different hexagonal structures. A hexagonal cluster consists of cluster members (CMs) and a cluster head (CH). The CHs are selected from the CMs based on nodes near the optimal CH distance and the residual energy of the nodes. Additionally, the optimal CH distance that links to optimal energy consumption is derived. To balance the energy consumption and the traffic load in the network, the CHs are rotated among all CMs. In WSNs, energy is mostly consumed during transmission and reception. Transmission collisions can further decrease the energy efficiency. These collisions can be avoided by using a contention-free protocol during the transmission period. Additionally, the CH allocates slots to the CMs based on their residual energy to increase sleep time. Furthermore, the energy consumption of CH can be further reduced by data aggregation. In this paper, we propose a data aggregation level based on the residual energy of CH and a cost-aware decision scheme for the fusion of data. Performance results show that the CCBE scheme performs better in terms of network lifetime, energy consumption and throughput compared to low-energy adaptive clustering hierarchy (LEACH) and hybrid energy-efficient distributed clustering (HEED).

## 1. Introduction

Typical sensor nodes are able to carry out sensing, data processing and communicating components, making them feasible for a wide range of promising applications, such as: environmental monitoring (e.g., humidity, temperature), disaster, healthcare, military, *etc.* [1]. Sensor nodes for these applications are usually deployed densely and operated autonomously. Sensor nodes are normally battery powered and left alone in adverse environments, making it quite challenging to recharge or replace node batteries. Hence, one of the crucial challenges in WSNs is to prolong network lifetime due to constrained energy resources. The critically-located sensors are those located near the sink, which carry the burden of relaying large amounts of data traffic, especially when multiple high-rate routes pass through these nodes. Thus, avoiding the failure of such nodes caused by early energy depletion is critical for improving the network lifetime. Another important challenge occurs, when each and every node wants to simultaneously transmit and receive data at the same time. This will lead to a lot of data collisions and congestion. As a result, a large amount of energy is wasted, and most of the nodes will run out of energy very quickly. Many proposals have concentrated on improving the energy efficiency. The research in data-centric WSNs is concentrated on clustering by reducing the number of transmissions to the sink, by selection of a proper MAC layer and an energy-efficient data aggregation mechanism to alleviate the challenges of WSNs.

Clustering means grouping the nodes based on geographical location into different clusters. The clustering technique decreases the number of nodes required to forward data to the sink node, thereby reducing the channel load and improving the scalability. However, the advantages of clustering algorithms comes with additional overheads during CH selection and the cluster formation process. Moreover, some CHs die early as compared to the other sensor nodes. These challenges can be alleviated by using the rotation of CHs and selecting proper MAC protocols. Improvement in the MAC layer can increase the reliability and increase the sleep time of nodes. The MAC layer can be based on contention, contention-free or hybrid protocols. Specifically, using contention-based protocols, such as carrier sense multiple access with collision avoidance (CSMA/CA), can result in a long contention among nodes for using network resources [2]. Besides, the performance of CSMA/CA decreases dramatically when the network load and node density increases. Contention occurs when nearby sensors attempt to access the wireless channel at the same time. Contention increases packet dropping, which decreases the reliability of network. As a result, contention-based MAC protocols are not suitable for sensor networks with high density. In order to alleviate this issue, several contention-free protocols, such as TDMA, are proposed, where time is divided into transmission slots that are assigned to CMs, according to a slot scheduling algorithm in CH. Improving TDMA slot allocation by increasing sleep time could potentially lead to the improvement of the reliability and energy efficiency of WSNs. TDMA is suitable for real-time applications, and convergecast is a widely-used communication technique where CH collects information from CMs and forwards to the sink.

Energy efficiency can be further increased by data aggregation algorithms in WSNs. Data aggregation (DA) techniques enhance the network lifetime by gathering and aggregating the data in an energy-efficient manner. The purpose of DA is to collect the useful information by eliminating repeated readings of a sensor. Aggregation reduces the communication cost by improving the energy consumption and network lifetime of the WSN. DA minimize the congestion by reducing the packet size.

In this paper, we propose the cross-layer cluster-based energy-efficient (CCBE) algorithm based on the combination of clusters, hybrid MAC and data aggregation. In order to minimize energy consumption, we derive the optimal CH distance, which links to optimal energy consumption. The CCBE algorithm divides the entire sensor nodes in the network to different hexagonal clusters. The CMs of each hexagonal cluster elect a CH based on optimal CH distance and the residual energy of the nodes. The CMs of each cluster are given time slots based on their residual energy in order to increase the network lifetime and synchronize the sleeping period. Furthermore, the energy consumed for the transmission of larger packets is reduced by data aggregation in CHs. The data aggregation is achieved by the fusion of similar data. The decision to fuse data is based on the cost-aware decision scheme introduced in this paper. Additionally, the aggregation level of CH is based on the remaining energy.

The remainder of the paper is organized as follows. In Section 2, we continue with related work. Section 3 shows the energy consumption model. In Section 4, we derive the optimal CH distance. In Section 5, we propose the new scheme CCBE. Section 6 shows the performance of the proposed scheme, and we provide conclusions in Section 7.

## 2. Related Work

This section provides a brief overview of existing energy-efficient techniques in WSNs. Different techniques have been carried out to design feasible protocols that improve energy efficiency in WSNs. In WSNs, scalability, reliability and predictability are considered to be major problems in high density networks. Clustering is considered to be the best solution for improving scalability. Energy efficiency is crucial to prolong the network lifetime of WSNs. Moreover, the selection of the MAC protocol is considered important for predictable channel access and reliability [3]. Furthermore, data aggregation improves the reliability and scalability for high density networks. In this paper, we are concerned about cluster formation, CH selection schemes, MAC and data aggregation in CHs.

In clustering, the nodes are grouped into different clusters, and each cluster contains one CH that forwards the data to the sink node or to the nearest CH. Many clustering algorithms are proposed to increase network lifetime among the nodes in the WSNs. Some of the well-known clustering algorithms are low-energy adaptive clustering hierarchy (LEACH) [4], energy efficient clustering scheme (EECS) [2], energy-efficient unequal clustering (EEUC) [5], *etc*. In LEACH [4], the CHs are elected based on the minimum communication distance to the maximum number of one-hop neighbors. Another feature is that CH will be rotated among the members of the cluster. The CH re-election and cluster formation happens every round. Unfortunately, this will lead to additional delay and energy usage.

HEED [6] selects CHs based on the remaining energy of sensor nodes. All other sensor nodes join the CH based on the minimum communication cost between CM and CH. In the second round, the election of CHs is based on two parameters: communication cost between CMs and remaining energy. However, the hot spot issue appears in areas that are close to the sink, as nodes in such areas need to relay incoming traffic from other parts of the network. The energy of these nodes gets depleted early as compared to other nodes.

The authors of [2] proposed EECS, which selects the formation of the cluster based on the weighted parameter to minimize the energy used for communication to the sink node. This is achieved by allocating clusters who are far away from the sink with a small number of CMs. Simulation results show that EECS performs better compared to LEACH in terms of energy efficiency. However, additional overheads are required for having information about other CHs and the sink node. Furthermore, it is not suitable for high density networks where nodes are scattered non-uniformly.

In EEUC [5], the cluster sizes with a longer distance from the sink node are larger compared to the cluster sizes near the sink node. This was proposed to save energy in communication between CMs and CHs. However, extra overheads are caused due to the additional data aggregation in all nodes, which degrades the efficiency of the network in a multi-hop environment. Furthermore, the energy of CH closer to the sink node tends to deplete quickly, because they forward more data traffic compared to other sensor nodes. The trade-off between the distance to the sink from the source and the cluster size should be studied analytically before setting up the network hierarchy.

The multi-hop routing protocol with unequal clustering (MRPUC) [7] was proposed to alleviate the hot spot problem. A sensor node that has the maximum remaining energy is elected as the CH. The CHs closer to the sink node have smaller cluster sizes to reduce the energy for heavy inter-cluster transmissions. The operation of MRPUC consists of three phases: cluster creation, routing between CHs and the sink node and packet transmission. However, inter-cluster multi-hop routing formation may cause an additional overhead.

In [8], the CH is elected based on the value assigned by the weighted factor to the CMs in the cluster. A new cluster formation is started when the value of the original CH drops below a certain threshold. Hierarchical cluster control (HCC) was proposed to improve various parameters in clustering. These are cluster size, connectivity between CHs and cluster joining. However, the entire network must be traversed before it can be computed.

In an emergent algorithm for highly uniform cluster formation (ACE) [9], one way of CH selection is when many nodes in its neighborhood do not belong to any cluster, then it elects itself as a CH. The two functional components of ACE are the generation of new clusters and the migration of existing ones. ACE uses a protocol to cluster the WSNs into a constant number of iterations. Unfortunately, it is hard to detect the number of iterations required to cluster WSNs. Moreover, additional overheads are caused by the migratory mechanism in ACE. In brief, total energy consumption in multi-hop data delivery in clustered WSNs should be analyzed comprehensively.

Several MAC protocols have been proposed by various researchers to reduce the energy consumption during the idle listening state. The energy efficiency is achieved by allowing sensor nodes to enter the sleep state periodically or aperiodically [10,11]. These MAC protocols are either based on contention, contention-free or hybrid approaches. Some of the recently proposed contention protocols, such as Short Preamble MAC Protocol (X-MAC) [12] and Low Power Down link MAC Protocol (WiesMAC) [13], improve the energy efficiency by minimizing the length of the listening state and duty cycle. These are achieved by using an adaptive preamble sampling scheme [14]. This scheme can reduce the energy used for transmitting packets. However, WiesMAC consumes more energy in multi-hop communications. In X-MAC [12], the energy efficiency can be achieved at both ends of data communications. This energy efficiency is achieved by using a shortened preamble sampling and an adaptive duty cycle for different channel loads at the receiver. However, the simulation results of X-MAC are not good enough to prove the effects of the algorithm.

Contention-free protocols, such as energy-efficient MAC protocol (S-MAC) [10], adaptive energy-efficient MAC protocol (T-MAC) [15] and utilization based duty cycle tuning MAC protocol (U-MAC) [16], can specify when nodes are awake and asleep within a period. These contention-free MAC protocols can be divided into periodic and event-driven based on the frame. Some of the periodic protocols are [10,15,17,18]. The nodes in these protocols synchronize the frame and time slots with each other. Additionally, nodes periodically wake up, transmit and receive messages. Then, nodes go to sleep until the next period to minimize energy consumption. However, the length of the listening state is not considered.

In T-MAC [15], the authors shortened the length of the listening state. Then, nodes go to the sleep state if no data are received during this time period. However, it will not converge in the case of event-driven traffic. U-MAC was proposed to solve this problem by tuning the duty cycle according to traffic load. However, some nodes may have higher data traffic in a frame considered with respect to other nodes. These nodes will have less sleeping time due to the periodic frame. Event driven TDMA Protocol (ED-TDMA) [19] alleviates this problem by changing the length of the TDMA frame. The length of the frame depends on the number of source nodes. However, if a CM needs more than one time slot to transmit data, the CH cannot allocate more than one time slots in one frame. Wu *et al.* [20] propose a distributed TDMA scheduling protocol based on coloring algorithm (TDMA-CA) utilizes spatial reuse of the wireless channel. The TDMA-CA protocol assigns distinct colors for conflict sensors in the WSNs and arranges different slots for data transmission for each color. However, additional overheads are created for synchronization among all nodes in the network.

In research of data aggregation schemes, there are different methods of data aggregation based on tree- and cluster-based aggregation. The tiny aggregation service (TAG) algorithm [21] is based on a tree structure network, where the number of transmitted packets are decreased by data aggregation. Cluster-based aggregation is used to overcome the problem of transmission delay and the loss of data due to node failures in the route to the sink. The CH aggregates the information from CMs and performs the average, or minimum, or maximum aggregation function. Different cluster-based protocols are explored in [22]. In cluster-wide correlated grouping (CWCG) [23] hybrid structure for data aggregation is proposed, which uses the concept of the temporal and spatial grouping of nodes. It provides reduced transmission cost, but increases the latency. In adaptive data aggregation (ADA) [24], the temporal aggregation degree is controlled by the reporting frequency of the events at the sensor nodes, and the spatial degree of aggregation is set by the aggregation ratio at the CH. The sink has the central control for aggregating the data by sending the temporal and special degree. It provides improvement in the desired reliability compared to that observed.

In [25], a grid-based spatial correlation clustering algorithm (GSCC) forms the sensor nodes with high similarity of data values in the same clusters. After the cluster formation, the cluster head node, which is located nearer to the center of the grid, approximates the data of the cluster members according to historical data, and then, only the cluster head sends the aggregated data to the sink. This algorithm is based on data with a Gaussian distribution.

In this paper, we consider all three areas of clustering, MAC and data aggregation. We propose a CCBE protocol that has three phases. In the setup phase, it calculates the optimum size of the hexagonal structure and divides the entire network nodes using this structure into different clusters. Then, it selects the CH based on the optimum CH distance and residual energy. In the slot allocation phase, the sink node assigns time slots to the CHs, and CHs assign time slots to the CMs based on the energy capacity. Thirdly is the steady transmission phase, where the CMs send the messages to CHs and CHs gather and aggregate the data using various decision schemes. After aggregation of information, it then forwards to the sink node.

## 3. Energy Consumption Model

In this paper, we use a radio model proposed in [4]. This radio energy model is utilized to measure energy consumption for the proposed CCBE algorithm. In [4], the radio model is a combination of three main models: the transmitter, the receiver and the power amplifier. The energy consumed by the transmitter consists of transmitter circuitry and the power amplifier, and the energy consumed in the receiver for receiving data consists of the receiver circuitry [4]. When a packet is transmitted from a transmitter to a receiver, where the distance between them is *d*, the received signal power at the receiver is [26]:

(1) $$p_{r}\left(d\right)=\frac{p_{t}G_{t}G_{r}\lambda^{2}}{{\left(4\pi \right)}^{2}d^{\beta }\text{Loss}}$$

where *G_r_* and *G_t_* are respectively receiver and transmitter gains. Furthermore, *Loss* represents any additional losses in the packet transmission, and λ represents carrier wavelength. The propagation loss factor β typically varies between two and four. Hence, considering *G_t_* = *G_r_* = 1, and *Loss* = 1, the received signal power at the receiver is:

(2) $$p_{r}\left(d\right)=\frac{p_{t}\lambda^{2}}{{\left(4\pi \right)}^{2}d^{\beta }}$$

To receive the data packets successfully, the received signal power at the receiver must be above a minimum threshold power (*p_thr_*). Hence, the transmitter signal power at the transmitter must be above this threshold $\frac{p_{\text{thr}}{\left(4\pi \right)}^{2}d^{\beta }}{\lambda^{2}}$. The energy absorbed by the transmitter is:

(3) $$E_{t}=(e_{e}+\frac{p_{\text{thr}}{\left(4\pi \right)}^{2}R^{\beta }}{\lambda^{2}d_{r}})\times \text{Packet}=(e_{e}+e_{a}R^{\beta })\times \text{Packet}$$

where $e_{a}=\frac{p_{\text{thr}}{\left(4\pi \right)}^{2}}{\lambda^{2}d_{R}}$, which is considered as the energy/bit absorbed in the transmitter RF amplifier, *d_R_* is the transmit or receive data rate (*bit*/*second*) of each network node, *Packet* is the number of bits in the packet and *e_e_* is the *energy*/*bit* consumed in transmitter electronics. Hence, based on [27], the energy absorbed per second by a sensor node in three states can be calculated as follows:

(4) $$\begin{array}{l} E_{t}=(e_{e}+e_{a}R^{\beta })N_{t}\\ E_{r}=(e_{e})N_{r}\\ E_{l}=e_{l}T_{l}=e_{l}(1-T_{t}-T_{r})=e_{e}d_{R}(1-T_{t}-T_{r}) \end{array}$$

The time for receiving and transmitting the data traffic between the cluster is denoted by *T_r_* and *T_t_*, respectively, where *N_t_* and *N_r_* are the traffic data bits transmitted and received, respectively Equation (5) represents the value of *T_r_* and *T_t_*.

(5) $$\begin{array}{l} T_{t}=\frac{N_{t}}{d_{R}}\\ T_{r}=\frac{N_{r}}{d_{R}} \end{array}$$

The amount of time spent in one second for listening to the radio channel is represented as *T_l_* : *T_l_* = 1 − *T_t_* − *T_r_*, (0 ≤ *T_l_* ≤ 1); thus, $0\leq (1-\frac{N_{t}}{d_{R}}-\frac{N_{r}}{d_{R}}\leq 1)$. Considering the static data traffic environment, where *N_t_* = *N_r_* = *N*, the value of *N* is represented in Equation (6) as:

(6) $$0\leq N\leq \frac{1}{2}\times d_{R}\times 1\text{second}$$

As a result, $\frac{1}{2}\times d_{R}$ bits represents the maximum amount of data that can be transmitted in each cluster per second, when nodes in the cluster do not listen to the radio environment and spend half a second for receiving the packets and another half for transmitting the packets. In simulations, we consider *d_R_* = 2.5 × 10^5^ bps [28]; the maximum data that can be relayed in WSNs based on Equation (6) is 1.25 × 10^5^ bits per second, where the energy consumed for listening to the radio environment per second is represented as *e_l_*. *e_e_*, *e_a_* and *e_l_* are obtained from the design characteristics of the transceivers. From [4,28], the specific values of *e_e_* is: *e_e_* = 3.32 × 10^−7^ J/bit. When *p_thr_* = 2 × 10^−9^*w*, *d_R_* = 2.5 × 10^5^ bps, *f* = 2.4 × 10^9^ Hz, and *e_a_* ≈ 8 × 10^−11^ J/bit/m^2^.

## 4. Optimal Cluster Head Distance

**Figure 1:**
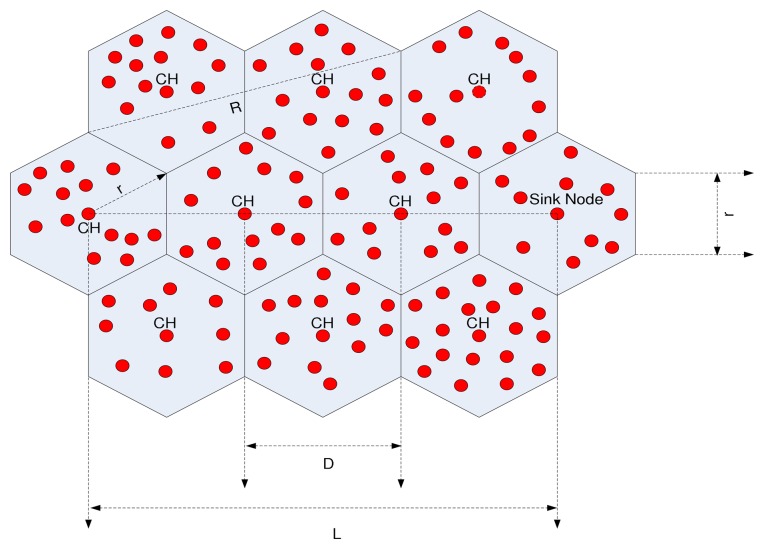
Cluster-based end-end multihop transmission in a hexagonal structure.

In an end-to-end multi-hop transmission considering an equal hexagonal cell, the best route between the source and the sink node is the direct line between them, where intermediate nodes are properly deployed (the nodes exists whenever needed). As shown in Figure 1, the data packet is transmitted from the source to the sink node, where *L* is the distance between them. Assuming that the distance between each CH is *D*, *m* is the number of CHs and is derived as:

$R=\sqrt{13}\cdot r$, $D=\sqrt{3}\cdot r$,

(7) $$m=\frac{L}{D}=\frac{L}{\sqrt{3}\cdot r}$$

In this paper, we consider static traffic in the network. Network is considered to have static traffic when the traffic rate following along the network is constant. In the hexagonal cluster model, *r* represents the side of the hexagon and the optimal CH distance, while *R* is the maximum transmission range of a node and the range should be such that two nodes located anywhere in adjacent cluster should be able to transmit and receive data. The energy consumed for the end-to-end multi-hop transmission based on the energy consumption model discussed earlier is:

(8) $$\begin{array}{ll} E_{i} & =E_{t}+E_{r}+E_{l}\\ & =\left(e_{e}+e_{a}R^{\beta }\right)N+\left(e_{e}\right)N+e_{e}d_{R}\left(1-\frac{N}{d_{R}}\right)\\ & =\left(2e_{e}+e_{a}R_{i}^{\beta }\right)N \end{array}$$

(9) $$\begin{array}{ll} E_{t} & =m\cdot E_{i}\\ & =\frac{L}{\sqrt{3r}}\left[2e_{e}+e_{a}{\left(\sqrt{13r}\right)}^{\beta }\right]N \end{array}$$

In order to find the minimum energy consumption, we take the first derivative of *E_t_* with respect to the optimal CH distance, *r*, and let: $\frac{d}{dr}\left(E_{t}\right)=0$

(10) $$\frac{d}{dr}(E_{r})=\frac{L}{\sqrt{3}}(e_{a}N{\left(\sqrt{13}\right)}^{\beta }(\beta -1)r^{\beta -2}-\frac{2e_{e}N}{r^{2}})$$

From Equation (10), we can derive the value for *r*, the optimal CH distance, as:

(11) $$\begin{array}{ll} r & =\frac{1}{\sqrt{13}}{\left(\frac{2e_{e}}{(\beta -1)e_{a}}\right)}^{1/\beta }\\ & =\frac{1}{\sqrt{13}}{\left(\frac{2e_{e}\lambda^{2}d_{R}}{(\beta -1)p_{\text{thr}}{\left(4\pi \right)}^{2}}\right)}^{1/\beta } \end{array}$$

**Figure 2:**
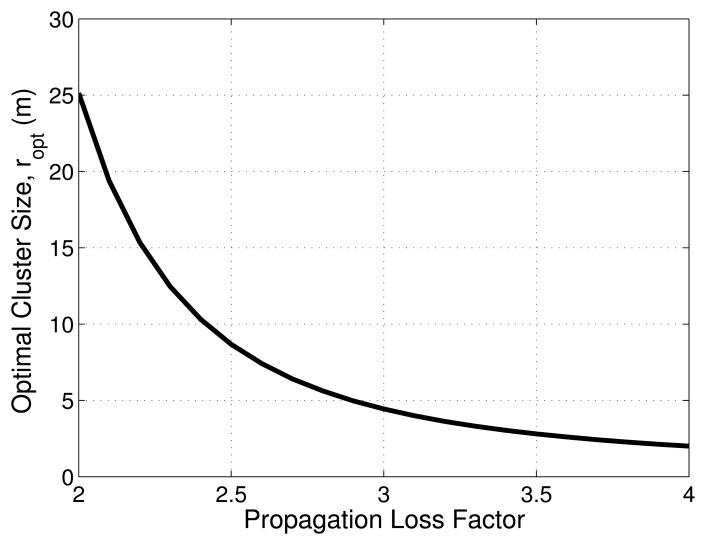
Optimal cluster head (CH) distance *r* variation with β.

By using correct transceiver parameters, Equation (11) shows the optimal CH location *r*, which depends on the propagation loss factor β with values ranging from two to four and with the network traffic, and the relationship between propagation loss factor β and *r* is shown in Figure 2. In Figure, the optimal CH distance and the side of the cell decrease when the propagation loss factor β increases, while network traffic *N* is constant. Furthermore, there is a trade-off between the number of CHs and the energy consumed in each CH. Based on Equation (8), when the number of CHs increases, the length of the side of the hexagonal cluster decreases, then energy consumption is dominated by the fixed energy consumption of each CH. When the number of CHs decreases, the length of the side of the hexagonal cluster increases, and energy consumption is dominated by the CH, because the energy consumed in the transmitter amplifier of each CH increases quickly.

## 5. Cross-Layer Cluster-Based Energy-Efficient Protocol

In this section, we explain the procedure of the CCBE that is proposed in this paper. The CCBE employs the self-organization technique for clustering of WSNs. In the proposed scheme, each node has to perform the basic task of sensing the field parameters, form data packets, and to communicate with the CH. Clustering in WSNs means partitioning nodes in a network into different clusters. The network model considered in this paper is a hexagonal structure [29], shown in Figure 1 with sensor nodes and the sink node. The sink node is constant and fixed for each simulation. Sensor nodes are homogeneous in nature, are assigned with a unique identifier and have the same capability. They are able to switch between active and sleeping states. CH nodes can forward the collected messages to their next hop CHs in the direction of the sink nodes. In CCBE, each node shares information about the current energy state, location, cluster-ID and the CH-ID with its one hop neighbors. The nodes of CCBE will be in four different modes. The four modes are described as follows.

- Cluster head (CH): While in CH mode, it broadcasts messages claiming its election to CMs. The CH then allocates time slots to different CMs registered in its database. The CH gathers and aggregate information from its CMs. Thus, they are responsible for conveying the complete information of their CMs. CHs are responsible for gathering, aggregating and forwarding the data to the sink or optimal distance CH in the direction of the sink node. CH sends or receives messages between the adjacent CHs or to the sink node at regular intervals using the assigned time slot.
- Cluster member (CM): A CM is a member that belongs to a particular cluster; it regularly transmits the collected information to its CH.
- Dead node: This is a state in which the sensor node cannot operate anymore, because its energy has been depleted completely or it has broken down. The node can neither transmit nor receive the data. In addition, the node is considered to be in this state when its residual energy (*E_res_*) is below 0.05 J.
- Isolated node: This means node does not have any one-hop neighbors, either to transmit or receive data, or it does not receive any CH messages.

The CH re-election happens only when the current CH value falls below the threshold value. Clustering solutions can be combined with TDMA-based schemes to reduce the cost of idle listening. CCBE is divided into the setup, slot allocation and steady transmission phase.

### 5.1. Setup Phase

The first phase is the setup phase, where sensor nodes are classified into certain clusters based on a hexagonal structure. The optimal length of each cluster side for β = 2 is found from Figure 2. Then, the cluster is created using the optimal cluster side for mitigation of the hot spot problem and to balance energy consumption. The formation of a hexagonal cluster with equal length cluster sides will help to choose CHs closer to the optimal CH location. However, initial CHs are elected randomly from CMs in the cluster. Then, the CH broadcasts the CH election message to all one-hop neighbors; those sensor nodes that receive these messages update its CH status.

(12) $$\begin{array}{cc} \overset{N}{\underset{j=1}{\text{∑}}}\frac{R_{ij}}{N}=r^{i} & \forall i=\left[1,N\right] \end{array}$$

(13) $$f(E_{\text{res}},r^{i})=\alpha \frac{r^{i}}{2r}-(1-\alpha )\frac{E_{\text{res}}}{E_{\text{cap}}}$$

CH re-election happens when the current CH value falls below a particular threshold. During the re-election, the CH is decided based on the remaining energy and optimum CH distance of the node to reduce the energy consumption and increase the network lifetime. Each CM in the cluster finds the value of Equation (12), where Equation (12) N is the number of the one-hop neighbors of the sensor node, *R_ij_* is the distance between node *i* and node *j*, *r^i^* is the average distance from node *i* to all its one-hop neighbors. Different CMs have different *r^i^* values. All CMs broadcast an advertisement message with their residual energy, average distance from each CM to all their neighbors, the location, the cluster-ID and the CH-ID using CSMA/CA.

The CHs are selected by a rule of best candidate, which selects a sensor node closer to the optimal CH location with minimum distance to all neighbors and has maximum remaining energy as a CH for the next round. The CCBE algorithm decides the CH node as the node (within the cluster) that minimizes Equation (13). In Equation (13)*r^i^* is the average distance to all neighbor nodes and *E_res_* is the remaining energy of the node. Intuitively, without taking energy balance into account, some sensor nodes may be selected more frequently as the CHs, and the energy of these nodes may be depleted very quickly compared to other member nodes. Since *r^i^* and *E_res_* use different units, they should be normalized, and Equation (13) shows the normalized form. The default energy capacity of each node (*E_cap_*) is used to normalize *E_res_*. *r^i^* is normalized with respect to the maximum distance between two nodes 2*r* in the cluster. The weight function *α* determines the relative importance placed on these two parameters. Moreover, this method is an effective way to the optimal node as the CH. Different applications have different requirements, and these weighting factors can be varied accordingly. This means that the nodes are decided to be the CH depending on the combined remaining energy (*E_res_*) of the node and the optimal CH location with the minimum average distance to all neighbors. This node uses the best combination of the minimum energy needed to reach neighbors and with maximum residual energy. Therefore, CCBE is higher in concept and in terms of energy efficiency. However, if the current CH dies for some unexpected reason, the CM with the next highest value will take over the previous CH's role.

### 5.2. Slot Allocation Phase

In the slot allocation phase, CH synchronization is required between other CHs, CMs and the sink node after its election. Moreover, CMs will have an additional listening state, so that adjacent CMs can synchronize their activities. During each contention period, all CMs keep their radios turned on. Here, CH will use a short preamble (similar to X-MAC [12]) to synchronize the CMs. In other words, when a new CH is elected, it will broadcast a SYN message, which contains its ID and the cluster-ID. After receiving the SYN message, the CM will send ACK messages to the CH. After receiving **ACK** message, the CH starts allocating the time slots based on residual energy.

In this paper, we propose channel allocation based on contention-free communication to reduce the energy consumption. CCBE logically divides the time into slots with slot size *t_s_*. A frame length *T* is composed of consecutive time slots. The activities of every node are then repeated with period *T*. We consider that all CHs have the same frame length. When a CM is transmitting packets to a CH, some other CMs that are in the listening state will also consume energy. Therefore, to reduce the total energy consumption, the other CMs that are not transmitting to CHs will go to sleep. The number of time slots for CMs is equal to the maximum possible number of members in the cluster.

The CHs build a TDMA schedule and broadcast it to all CMs within the cluster. The scheduling algorithm assigns time slot 1 ≤ *t* ≤ *T* to all CMs. The members can be in any of the four possible states: transmitting, receiving, listening and sleeping. Considering that all CMs are perfectly synchronized and using TDMA, then no CM needs to be in the listening state during the transmission of other CMs of the same cluster. Our objective is to schedule the activities of sensor nodes to minimize the state transitions (especially from the sleeping state to active states) in order to increase energy efficiency. Surprisingly, the schedule for channel access is designed in such a way that CMs needs to wake up only twice: once for receiving information from their CHs and another time for transmitting their data to their CH. Moreover, consider a situation in which if CMs are scheduled in a random order, some CMs may need to wake up multiple times in a scheduling period T. This will increase the energy cost for each CM. Based on the new CCBE transmission schedule, each member turns on or off its radio. If a member has packets to send, the radios of all other members can be turned off. If the schedule indicates that a member node is a recipient in a certain time slot, its radio needs to be turned on.

**Figure 3:**
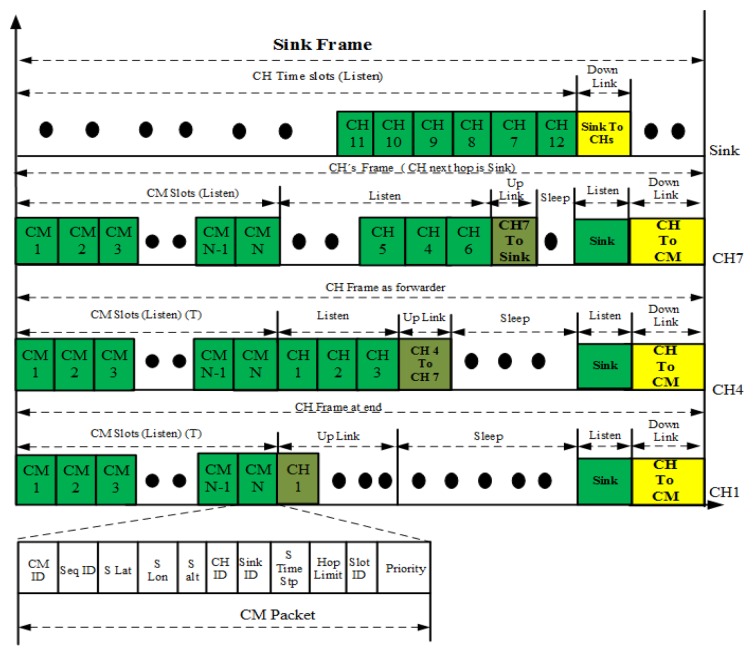
TDMA in both sink and CH frames.

TDMA is used by both CHs and CMs for accessing the wireless channel. The time for accessing the channel is divided into different slots. Moreover, a CH or CM has to achieve the right to use a slot before data transmission. In this protocol, the sink assigns time slots to CHs, and the CH assigns time slots to CMs. From Figure 3, the sink time frame is divided into CH time slots and sink down-links. The packet header of CM packets is shown in Figure 3.

The frame of CH as a starter (CH1), forwarder (CH4) and CHs' next hop as the sink (CH7) are shown in Figure 3. CH1 represents the CH of the cluster at the extreme end. Moreover, CH1 is not required to listen to other CHs for forwarding information to the sink. The frame of CH1 is divided into five slots. These are for listening to the CM and sink transmission, transmitting data to its next hop CH and its CMs and the sleep state. The CHs assign the time slot to CMs based on their remaining energy. The CM with the least energy will be given the first slot to transmit to the CH. The CM goes to sleep after transmission and only wakes up to receive the data from the CH in the same frame. Then, next is the CH forwarding state to the next hop CH in the direction of the sink. The sleep states of each CH are varied depending on the location of the CHs. The CHs at the extreme end, such as CH1, have more sleeping time compared to the CHs near the sink.

The times slots in the frame are given a light green color for listening; the up-link is given dark green; and the down-link is given a yellow color. The difference between CH4 and CH1 is that CH4 contains an extra listening state to receive the data of CH1. In the listening state for CH data, it only forwards three CHs' data due to the hexagonal structure. The sink frame consists of six CHs slots due to the hexagonal structure of the clusters. In the sink down-link, it sends information to all if the CHs in network by single-hop communication. The network will stabilize once all CMs get their time slot in the CH frame and all CHs get the time slot in the sink frame. It is assumed that, in the system, all packets have the same size and all time slots also have the same length, except the slot of the CH and the sink transmission slot.

### 5.3. Steady Transmission Phase

**Figure 4:**
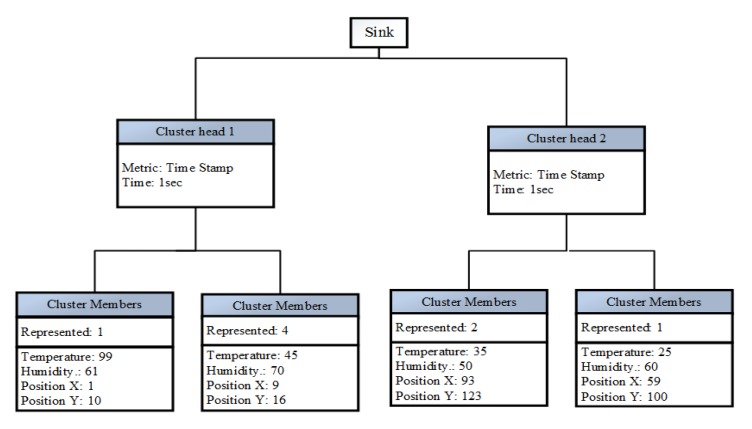
Aggregation based on clustering.

In the transmission phase, intra- and inter-cluster CH routing happens. In intra-cluster routing, the CM sends data to the CH in the same cluster. In inter-cluster routing, the data aggregated by the CH will be forwarded to the sink or CH node. The CH will forward the packets of three previous CHs to the sink according to the hexagonal structure. Before forwarding the data to the next CH or sink, it aggregates the data based on the cluster aggregation scheme. The cluster aggregation scheme consists of the sink, CHs and CMs. the cluster aggregation scheme is shown in Figure 4. The aggregation scheme can consist of different aggregation levels. The aggregation level of each CH is based on the remaining energy of the particular CH. More aggregation happens when the remaining energy is low. The energy window for an aggregation level is found by dividing the total energy capacity of the node by the total number of the aggregation level. The aggregation level increases when the energy depletion increases. The aggregation levels for different residual energy is shown in Table 1. The data aggregation has different components to reduce the data; for example, considering the temperature as a parameter, to fuse the temperatures of different sensor nodes. The goal of maintaining extreme data records is fusing many similar CMs and thereby maintaining as much precision of the data as possible. Thus, fusing similar objects is preferable. Most data aggregation schemes only focus on averaging data in the fusion process. In this paper, we propose a cost-aware decision scheme to select the two sensor nodes to fuse information to reduce the size of the packets. Furthermore, a cost-aware scheme helps to reduce the loss of extrema values and increases the precision of the data. Additionally, this aggregation level of each CH is used to control the amount of aggregation based on the current *E_res_* of each CH. The cost-aware decision uses a weight function to calculate the fusion costs of two CMs *a* and *b*, considering all contained parameters. Let *p* denote the set of parameters and *a_i_* be the value for the *i*-th parameter of node *a*. Furthermore, let *w_i_* be the weight for parameter *i*. Then, the cost can be calculated as indicated in Equation (14).

(14) $$\text{cost}=\sum_{i\in p}w_{i}*|\frac{a_{i}-b_{i}}{\max_{t}}|$$

(15) $$w_{1}+w_{2}+w_{3}\ldots +w_{i}=1$$

(16) $$w_{1}+w_{2}=1\Rightarrow w_{2}=1-w_{1},0\leq w_{1}\leq 1$$

Using this notation, assuming a system using only two parameters *P* = {*temp*, *hum*}, let the weights be 0.5 for both the temperature and position. The weights allow one to determine the importance of temperature and position. Furthermore, varying weights from zero to one can determine the best performance of the decision component.

For aggregation level, the total energy capacity of the CH and the number of aggregation levels are considered. The energy window (EW) size for each aggregation level can be calculated from Equation (17), where *E_cap_* is the energy capacity of sensor nodes and *n* is the total number of aggregation levels. This method can be explained further using an example; from Table 1, *n* is considered as 10 and *E_cap_* = 2*j*. The EW size is calculated, and each window size is assigned to each aggregation level. Firstly, each CH checks its *E_res_* and compares with the aggregation level size. If there is a change, then it updates its aggregation level. The CH checks its *E_res_* every time before it transmits to the sink node.

(17) $$EW=\frac{E_{\text{cap}}}{n}$$

After the aggregation of data in the CH, the CH will transmit data to the next CH in the direction of the sink. The CH operates at the range of *R*, which is the maximum distance between two adjacent cells in a hexagonal structure. In order to send neighbor information to all possible CH candidates in the direction of the sink, the CH will broadcast the next CH's signals. Furthermore, the CHs create a route towards the sink and select a final CH for relaying information to the sink. The “Final CH” is the CH node that has the next hop as the sink node. Forwarding packets through this route can reduce energy consumption compared to the direct transmission of messages from all CHs to the sink. During the creation of the route for inter-cluster routing, the CHs carry out their duties while transforming into the following three modes while relying on different roles.

- Initial mode: When the inter-cluster routing phase starts, all CHs are assigned with initial values during the initialization process.
- Route broadcasting mode: This is the mode where the signals are broadcast to establish an inter-cluster route.
- Route establishing mode: In this mode, routes are established from their own routes and those of their neighbors.

In this paper, we compare the CCBE algorithm with the LEACH [4] and HEED algorithm in terms of the network lifetime and throughput.

## 6. Simulation Results

### 6.1. Simulation Environment

We used ns-2.34 for performance evaluation of the proposed CCBE protocol. In our simulation environment, a network of 1800 nodes is deployed in an area of 400 m × 400 m with the sink in the center (200, 200) and the sink not in the center (350, 200). We set the initial energy of each node to 2 J. The area of one hexagonal cluster based on the optimum hexagonal side of 25.06 m is 1631 m^2^. The number of clusters created in the given area is 90 with the same number of CHs. There are 20 CMs in each cluster. The association of CMs with a particular cluster remains unchanged in the entire simulation. Only CHs change in the entire simulation. In this protocol, we consider periodic message transmission to the sink node. In the case of LEACH and HEED, 802.15.4 MAC is used to compare with the CCBE protocol. In this paper, we consider each node's energy consumption as the summation of energy consumed in the transmission and reception of data packets per round. The simulation parameters are given in Table 2, in which the parameters of the radio model are the same as those in [3]. We first obtain the optimal side length of the hexagon (*r*) from Equation (11) (*r* = 25.06 m for *n* = 2). We will examine the energy consumption and the maximum time delay with various numbers of nodes for CCBE, LEACH and HEED. Each CH is allocated a transmission slot in each frame of the sink node. According to the hexagonal structure, there are six neighboring CHs for the sink. These six CH nodes require a frame length of ω × 6 s, where ω is the slot duration of CH. At the beginning of each slot, nodes that are not transmitting go to sleep. The CHs aggregate the received data based on their residual energy and forward to the next CH or the sink node.

### 6.2. Performance Evaluation

We compared the CCBE algorithm for different values of *α* with the typical protocols LEACH and HEED by using various metrics: the energy consumption over simulation time, the fraction of alive nodes over time, the number of packets received at the sink, the maximum time delay with increasing number of nodes in the sensor network and the precision of the data received at the sink. The sink is placed at different locations of the sensor network to analyze the effect of location on energy consumption and network lifetime.

**Figure 5:**
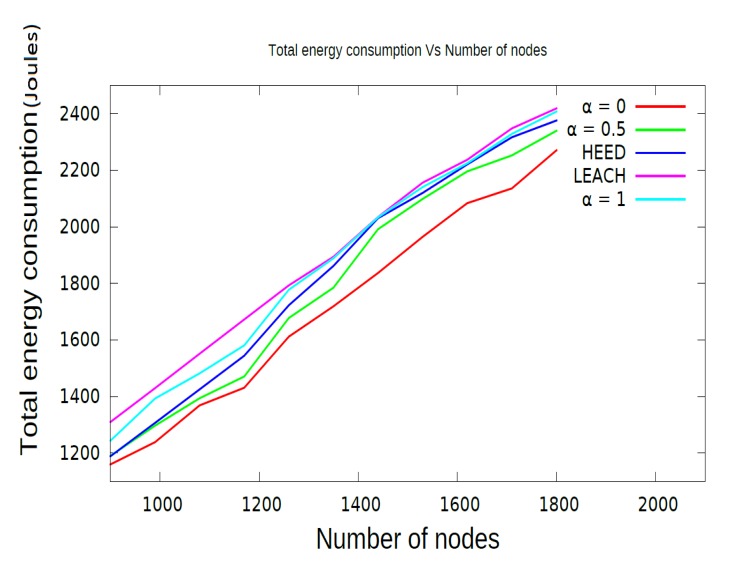
Comparison of energy consumption.

**Figure 6:**
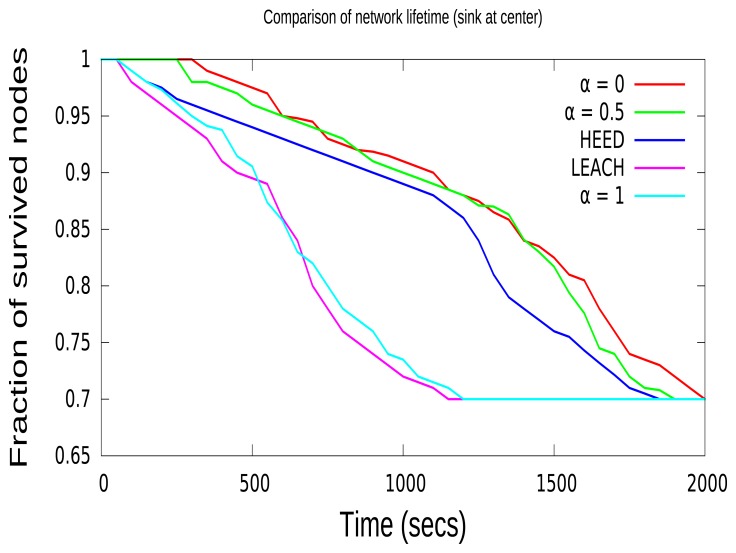
Comparison of network lifetime (sink in the center).

**Figure 7:**
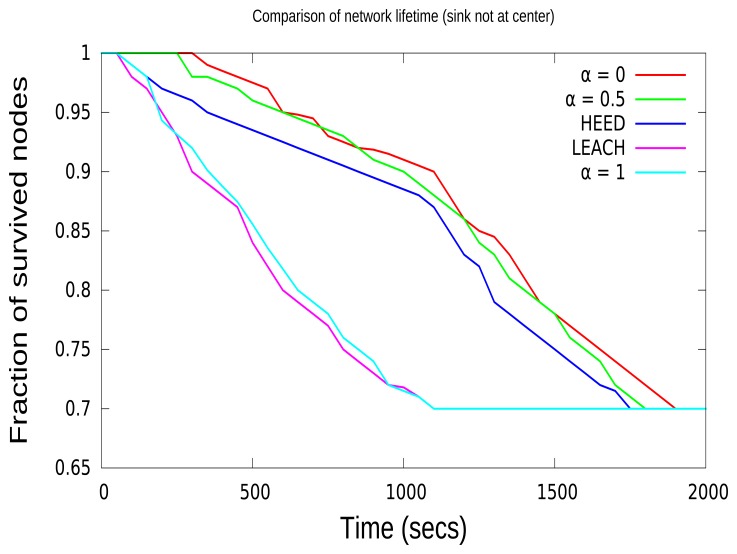
Comparison of network lifetime (sink not in the center).

The first performance metric, energy consumption over time, gives an idea of the rate of consumption of energy in the network. The initial energy given to each sensor node is two Joules. Figure 5 shows the variation of energy consumption with respect to the increasing number of sensor nodes in the network. As shown in Figure 5, the proposed CCBE (*α* = 0) protocol consumes less energy compared to other protocols, CCBE (*α* = 0.5), CCBE (*α* = 1), LEACH and HEED, respectively. CCBE (*α* = 0) consumes 64% of the energy of the entire network before 30% of the network nodes die. The energy consumption in CCBE is reduced by allowing the sensor nodes to sleep between the time for transmission and reception of packets. The energy consumption increases according to the increasing number of sensor nodes in the network for all algorithms. Less energy consumption means more network lifetime. This is analyzed using the network lifetime Figures 6 and 7.

The network lifetime is defined as the fraction of nodes alive over time. This gives an idea of the time over which the network can send the data before all of the nodes in the network die. When *α* is set to the optimal value, it will prolong the network lifetime; for instance, when the definition of the lifetime of the network is 30% of nodes completely depleting their energy. Additionally, 30% of nodes referred to above corresponds to a 70% “fraction of survived nodes” in Figures 6 and 7. This 30% of nodes does not have enough energy to receive or transmit a packet. In Figure 6, the network lifetime based on various values of *α* from zero to one is shown, when the sink node is in the center. In both Figures 6 and 7, CCBE (*α* = 1) and LEACH, the network lifetime decreases drastically compared to the other algorithms. This is due to the fact that LEACH and CCBE (*α* = 1) do not take into consideration the residual energy of nodes during CH selection. This may result in selecting CHs with nodes having less energy. This may further affect the early depletion of energy for a particular node and the loss of packets. CCBE (*α* = 0) considers only residual energy during CH selection, which results in having the highest network lifetime compared to other protocols. In CCBE (*α* = 0), nodes with lower energy are never selected as CHs when the cluster contains nodes with higher residual energy. Moreover, this reduces the packet loss in end-to-end communications. Both the HEED and CCBE (*α* = 0.5) select CHs based on both residual energy and communication costs. This results in having a moderate network lifetime compared to other protocols. The network with the sink node in the center has more network lifetime due to the reduction of the number of packets that need to be aggregated and forwarded to the sink.

In this paper, we define the lifetime of the network as the time duration before a fraction of nodes runs out of energy, and the results from Figure 6 show the efficiency of each algorithm when the sink is in the center. For instance, when the definition of the lifetime of the network is 30% of nodes completely depleted of energy, the ratio of the lifetimes of the network (calculated in loops between zero until 30 events) under various algorithms is as follows. LEACH : CCBE(*α* = 1) : HEED : CCBE (*α* = 0.5) : CCBE(*α* = 0) = 0.58 : 0.62 : 0.88 : 0.94 : 1; this shows that the CCBE algorithm (when *α* = 0, sink at center) has better performance in the network lifetime when analyzed with LEACH and HEED. Since LEACH and CCBE (*α* = 1) do not take into consideration the residual energy of nodes, the network lifetime decreases drastically compared to the other algorithms.

**Figure 8:**
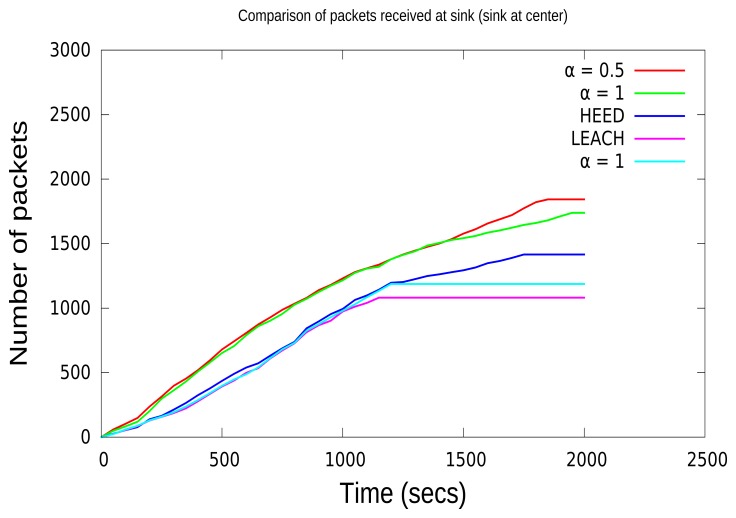
Comparison of data received (sink in the center).

**Figure 9:**
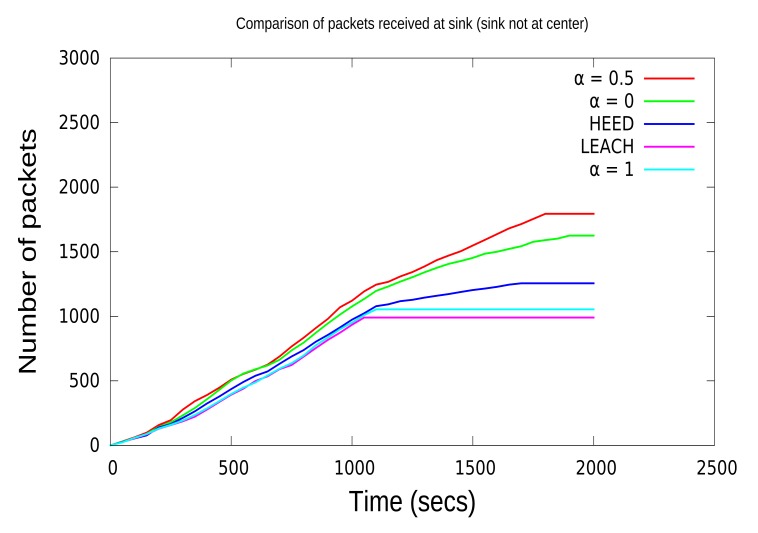
Comparison of data received (sink not in the center).

In order to analyze the effect of packet loss due to the selection of CHs with lower energy, in this paper, we have compared the number of packets received at the sink for various protocols. From Figures 8 and 9, it is clear that when the sink node is in the center, it receives a higher number of packets compared to the sink that is not in the center. Figures 8 and 9 show that before the network disconnection, the sink node of the CCBE (*α* = 0.5) algorithm receives much more data packets than the other algorithms. In Figure 9, the received data with CCBE (*α* = 0.5, 0) increase more quickly than the other protocols. Moreover, this is a result of using residual energy and the communication lost for CH selection. The number of packets received is shown in Tables 3 and 4 for both locations of the sink node. The sink node in the center for CCBE (*α* = 0.5) reduces the number of extra data transmissions from the CH to the sink node, thereby resulting in having a higher number of data packets received at the sink node.

**Figure 10:**
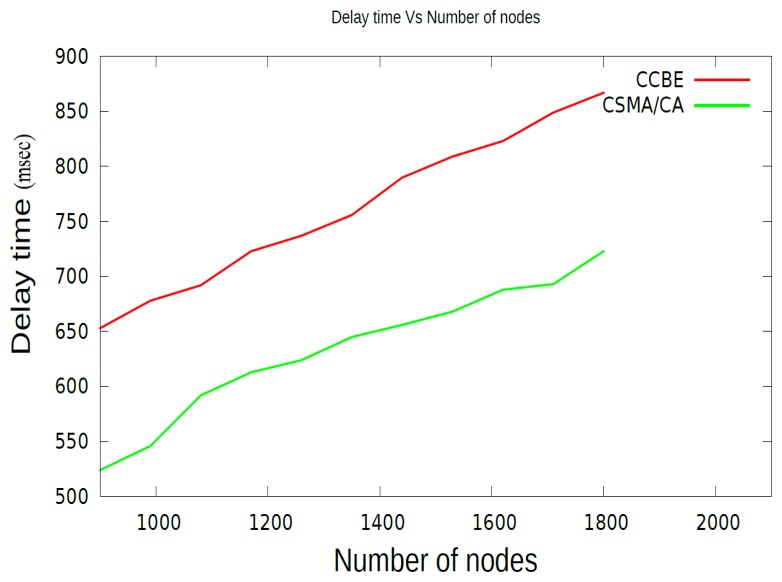
Comparison of the time delay.

In order to understand the performance of the MAC scheme proposed in this paper, the time delay with increasing number of nodes in the sensor network is shown in Figure 10. It is clear that the delay increases with the increasing number of sensor nodes in the network. In CCBE for all values of the *α* MAC scheme is based on TDMA. Moreover, the number of slots required also increases. This results in increasing delay for end-to-end communication. In Figure 10, we have increased the number of CMs in each cluster from 10 to 20. Figure 10 compares the delay for both CCBE and CSMA/CA, which is used in LEACH and HEED. CSMA/CA has less delay due to the random access of the channel, and in CCBE, the nodes need to wait for their time slot for transmitting the data to other nodes. However, the energy consumed during the idle listening time for each sensor node in CCBE is decreased by letting those nodes sleep till it is time for the transmission or reception of packets. The number of clusters remained the same, even though the number of CMs is increased in each cluster.

**Figure 11:**
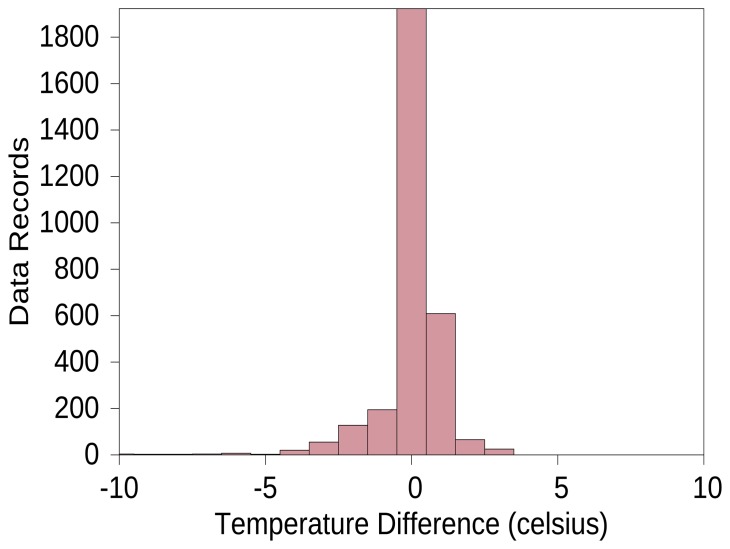
Precision of the data received.

The number of time slots for CMs in the CHs is increased. This will result in a higher packet size, so more energy is consumed in the packet transmission. The packets size is maintained by aggregating the data in the packets. Aggregating more data will result in more error or less precision at the sink node. Figure 11 shows the precision of data received at the sink node. The precision graph is obtained by comparing the values obtained at sink node with actual values sent by the CMs. In the simulations, each CM randomly sends temperature values to the CHs. From all graphs, it is clear that our protocol performs better compared to typical protocols, such as LEACH and HEED.

## 7. Conclusions and Future Work

In this paper, we proposed the CCBE algorithm to extend the network lifetime and to reduce the energy consumption in end-to-end packet transmission. The simulation results are compared with the well-known clustering algorithms, LEACH and HEED. The proposed CCBE algorithm divides all of the sensor nodes in the network into different hexagonal clusters. The side length of the hexagonal structure is derived, and clusters are formed. Then, the CMs elect a CH in the cluster. The CM (within the cluster) that minimizes the value of $\left[\alpha \frac{r^{i}}{2r}-(1-\alpha )\frac{E_{\text{res}}}{E_{\text{cap}}}\right]$ is elected as the CH. Furthermore, this CH is the node that has the best residual energy and requires the minimum energy to be reached by the CMs. In addition, weight parameter α decides the relative importance placed on these two parameters. The MAC scheme is based on TDMA. Sink nodes allocate the time slots to the neighboring six CHs. Then, the CH allocates time slots to all of the CMs based on the residual energy. The CMs change to the sleep state after the transmission and wake up only during the listening state (CH transmission to CMs). The CH collects data from three other CHs behind the current CH and forwards to the next CH or the sink. Before forwarding the data to the next CH, the CH aggregates the data based on the residual energy level and chooses the appropriate aggregation level. The results from simulations show that the CCBE algorithm has the best efficiency in terms of both data packets received by the sink node and the network lifetime.

CCBE creates the additional overhead of control packets during the end-to-end packet transmission and unbalanced utilization of nodes near the sink. Our next step is to compare the single-hop transmission from the CH to the sink and multi-hop transmission from the CHs to the sink node; additionally, to compare the energy consumed during data aggregation and to compare it without data aggregation; furthermore, to find the optimal location of the sink node based on the deployment of the sensor nodes; then, to adaptively increase and decrease the size of the hexagonal structure based on the number of alive nodes; moreover, to implement a dynamic traffic scenario with an adjustable hexagonal structure based on the cluster size.

## Figures and Tables

**Table 1:** Aggregation levels based on the residual energy of the CH.

Aggregation Level 0	2 <= *E_res_* < 1.8
Aggregation Level 1	1.8 <= *E_res_* < 1.6
Aggregation Level 2	1.6 <= *E_res_* < 1.4
Aggregation Level 3	1.4 <= *E_res_* < 1.2
Aggregation Level 4	1.2 <= *E_res_* < 1.0
Aggregation Level 5	1.0 <= *E_res_* < 0.8
Aggregation Level 6	0.8 <= *E_res_* < 0.6
Aggregation Level 7	0.6 <= *E_res_* < 0.4
Aggregation Level 8	0.4 <= *E_res_* < 0.2
Aggregation Level 9	0.2 <= *E_res_* => 0

**Table 2:** Parameters of the simulation.

**Propagation Factor (β)**	**2**
Network length	2000 m
Simulation time (*t*)	0.02 s
Number of nodes (*N*)	1500
Energy capacity of nodes (*E_cap_*)	2J
Optimal cluster size (*r*)	25.06 m
Min threshold power (*p_thr_*)	8×10^−15^ W
Carrier frequency (*f*)	2.4 × 10^4^ Hz
Data Rate (*d_R_*)	2.5 × 10^5^ bps

**Table 3:** Sink node in the center. LEACH, low-energy adaptive clustering hierarchy.

	***α*= 0**	***α* = 0.5**	**HEED**	**LEACH**	***α*= 1**
Lifetime (s)	1956	1835	1723	1132	1207
Packets	1739	1843	1416	1081	1187

**Table 4:** Sink node not in the center.

	***α*= 0**	***α*= 0.5**	**HEED**	**LEACH**	***α*= 1**
Lifetime (s)	1892	1756	1682	1021	1121
Packets	1625	1794	1256	990	1054
